# Cervical vertigo due to rotational fixation of atlantoaxial joint combined with benign paroxysmal positional vertigo: A case report and literature review

**DOI:** 10.1097/MD.0000000000039192

**Published:** 2024-08-02

**Authors:** Yahui Sun, Xingquan Wu, Huijuan Lou, Jinglei Jiang, Zhongxu Li, Jing Xu, Yiping Sun, Deyu Cong

**Affiliations:** a Department of Acupuncture and Tuina, Changchun University of Chinese Medicine, Changchun, China; b Department of Tuina, Affiliated Hospital of Changchun University of Traditional Chinese Medicine, Changchun, China; c Department of Tuina, Changchun University of Chinese Medicine, Changchun, China.

**Keywords:** Atlantic Rotary Fixation, benign paroxysmal positional vertigo, cervical vertigo, tuina

## Abstract

**Introduction:**

Vertigo is the most common clinical complaint, misdiagnosed patients are not rare, so it is very important to exclude and identify vertigo. For vertigo caused by multiple causes, including cervical vertigo with atlantoaxial rotation fixation combined with benign paroxysmal positional vertigo (BPPV), tuina can correct joint misalignment. The reduction technique will return the fallen otolith to the correct position. The use of massage and reduction can improve clinical symptoms and improve quality of life and may be a simple, safe, and effective treatment strategy for this disease.

**Patient concerns:**

We report on a patient with both cervical vertigo due to atlantoaxial rotational fixation and BPPV, including his imaging examination, clinical manifestations, and treatment methods.

**Diagnosis:**

Cervical vertigo (atlantoaxial rotatory fixation) and BPPV.

**Intervention:**

Tuina combined with atlantoaxial directional inverted reduction technique and reduction manipulation.

**Outcomes:**

The patient’s vertigo symptoms improved significantly, nystagmus disappeared, cervical occipital pain, nausea, head distension, and other symptoms disappeared, and cervical motion rotation reached 60°.

**Conclusion:**

This study proved the effectiveness of massage combined with a reduction in the treatment of cervical vertigo and BPPV, as well as the importance of vertigo diagnosis and differential diagnosis, and provided a new treatment idea for the future diagnosis and treatment of vertigo caused by a variety of causes.

## 1. Introduction

Studies have shown that vertigo is the most common clinical complaint, About 4% of patients in the emergency department and 5% in the outpatient department were treated for vertigo.^[1]^ In addition to relying on head computed tomography, magnetic resonance imaging (MRI), and other imaging techniques, it is also very important to ask about the cause and time of the disease and the characteristics of vertigo and to exclude and identify vertigo. Rule out life-threatening diseases, identify the cause, and quickly diagnose and treat them. Vertigo is considered by some to be the most difficult symptom to diagnose, and there is growing evidence that it is not uncommon for patients who come to the clinic with vertigo to be misdiagnosed.^[2]^ The manifestation of vertigo is different, and it may also be a symptom caused by a combination of multiple causes, Each cause needs to be explored, diagnosed, and the relevant treatment selected.

## 2. Case presentation

**Figure 1.**
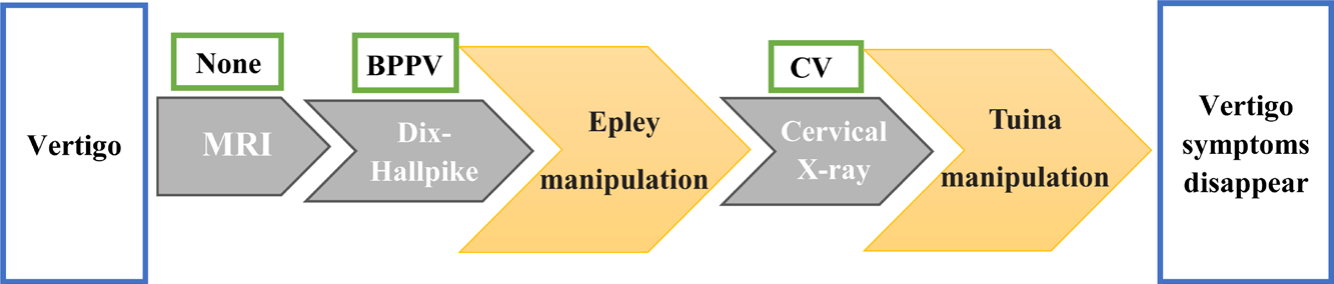
The flow of clinical and procedural data. BPPV = benign paroxysmal positional vertigo, CV = cervical vertigo, MRI = magnetic resonance imaging.

The flow of clinical and procedural data is shown in Figure 1.

The male patient, 65 years old, was admitted to the hospital mainly because of “Dizziness accompanied by neck pain for more than 1 month, aggravated for 7 hours” skewed quarrel. The patient reported dizziness with neck pain, tinnitus, and nausea after fatigue more than 1 month ago, and the symptoms worsened after turning the head. The results of the head MRI showed no obvious abnormality. Seven hours ago, the above symptoms worsened and she went to the undergraduate department. Patients present symptoms: persistent vertigo, accompanied by nausea, vomiting, and tinnitus, occasionally strong episodic vertigo, appearing in a fixed position, that is, from the sitting position to the right side when there is a “spinning” feeling, strong vertigo lasting about 1 minute. Physical examination: The patient had free movement of both eyes, large and round pupils on both sides, sensitive light reflex, normal tendon reflex, negative Hoffmann sign on both sides, difficulty standing test with closed eyes (-), blood pressure: 110/70 mm Hg. Combined with head MRI, other diseases of the central nervous system and brain were excluded. Dix-Hallpike test 3 was performed, which is an examination for benign paroxysmal positional vertigo (BPPV) in the vertical semicircular canal. The patient takes a sitting position, the examiner turns his head to one side 45°, maintains the head position, and quickly lies on his back, and the head is tilted back and overhanging at a 30° angle with the horizontal plane. At this time, the patient has vertigo symptoms of “spinning and spinning,” and then vertical torsional nystagmus and vertigo symptoms disappear for about 1 minute. Diagnosis: BPPV (vertical semicircular canal) was treated with Epley manipulation.^[3]^ After reduction, the patient lay on the right side, intense dizziness disappeared, Dix-Hallpike test (-), no nystagmus.

**Figure 2.**
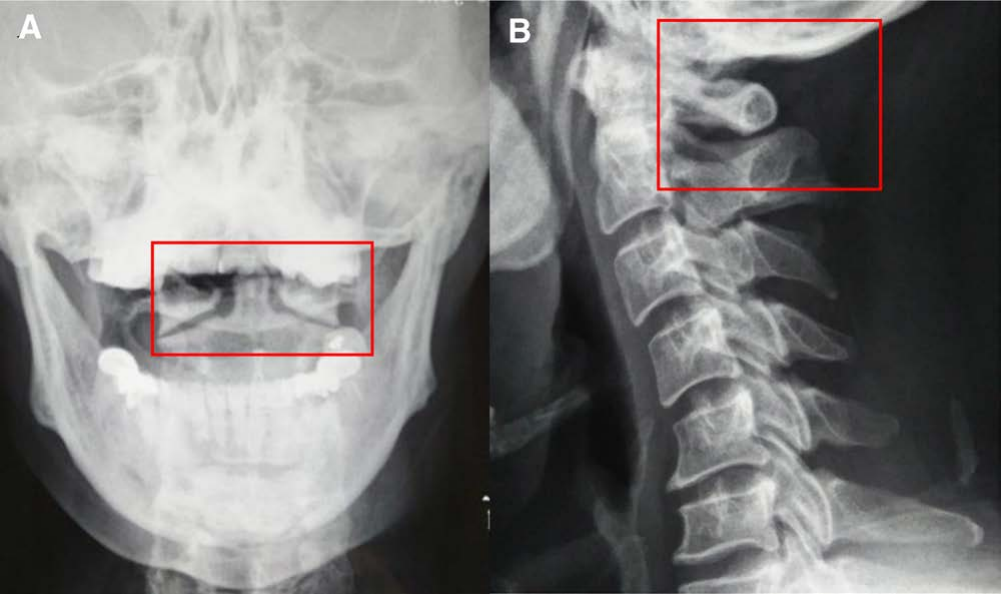
(A) Cervical open mouth X-ray (cervical open mouth X-ray); (B) lateral X-ray of the cervical spine (median sagittal section).

The patient reported persistent vertigo with cervical occipital pain, nausea, and head distension, which worsened during cervical flexion, extension, and rotation. Physical examination showed cervical occipital muscle tension, cervical rotation test (+), bilateral C1 transverse process tenderness (+), and supine neck rotation angle asymmetry: left rotation 20°, right rotation 30°. Open and lateral radiographs of the cervical spine showed that the physiological curvature of the cervical spine disappeared, the lateral mass on both sides of the atlas is unequal in size, and the atlas rotates clockwise relative to the axial vertebra, the spinous spaces of occipital, atlas and axial vertebrae were narrowed (Fig. 2). Vertebral artery color ultrasonography: bilateral vertebral artery distorted and tortuous, bilateral vertebral artery cervical rotation test (+), diagnosis: Cervical vertigo (CV)—atlantoaxial rotation fixation. Firstly, the spasmodic muscles around the neck were treated by tuina with release manipulation, and then the dislocation of the atlantoaxial joint was adjusted by directional elevation, then the tuina combined with atlantoaxial directional inverted reduction technique was used to adjust the atlantoaxial directional inverted reduction.^[4]^ The specific operations are as follows: First, rotate the head clockwise to the maximum angle, the left middle finger hooks the spine process to the left, the hypothenar supports the occipital, the right palm supports the mandibular, tilts up to the limit, do a slight upward flash. Then turn the head counterclockwise to the maximum angle, press the right side of the spine process with the right thumb of the right hand, support the occipital part of the thenar, support the mandible with the left palm, tilt up to the limit, and do a slight upward flash. After continuous treatment once a day for 5 days, vertigo symptoms improved significantly, and cervical occipital pain, nausea, and head distension symptoms disappeared completely. Physical examination showed that cervical occipital muscles were soft, cervical rotation test (-), bilateral C1 transverse process tenderness (-), and the rotation angles of both sides of the neck in supine position were symmetrical: left rotation 60°, right rotation 60°. Cervical motion: anterior flexion: 45°, posterior extension: 40°, left flexion: 40°, right flexion: 40°. Two months later, no dizziness and other symptoms, no recurrence.

## 3. Discussion

Vertigo can be divided into vestibular vertigo and non-vestibular vertigo according to the pathogenesis: vestibular systemic vertigo refers to the vertigo caused by the vestibular system, vestibular nerve, and vestibular central system. Non-vestibular vertigo refers to vertigo caused by vascular, systemic, or eye disease, such as cardiovascular disease, hypertension, hypotension, severe anemia, systemic toxicity, endocrine, head trauma, cervical spondylosis, and neurosis. Vestibular vertigo is divided into central vertigo and peripheral vertigo.^[5]^ Central vertigo refers to vertigo caused by lesions in the intracranial segment of the vestibular nerve, including the vestibular nucleus, suprachional fibers, medial longitudinal tract, cortex, and vestibular representative area of the cerebellum.^[6]^ Peripheral vertigo refers to vertigo caused by lesions in the inner ear portal, namely vestibular organs, vestibular neurons themselves, vestibular nerves, and receptor sites, including the inner ear labyrinth terminal receptors, the ampulla ridge of the semicircular canal, the elliptic sac, and the sensory spot of the balloon.^[7]^ Vertigo is a difficult disease to identify. In addition to the exclusion based on MRI, computed tomography, and other auxiliary examinations, a simple exclusion can be carried out at the stage of consultation and physical examination, so that clinicians can quickly and accurately assess the condition and exclude the cause of life crisis.

BPPV is the most common external vestibular dysfunction in clinical practice, with a reported 1-year prevalence of 1.6% and a lifetime prevalence of 2.4%.^[8]^ BPPV is characterized by a change in the position of the head in the direction of gravity that triggers episodic vertigo.^[9]^ Although the cause of BPPV is unknown and may be associated with head trauma, chronic reciting, and various causes of ear disease, BPPV is caused by free-floating otoliths that shed from the otoliths as a result of injury, infection, diabetes, migraine, osteoporosis, prolonged bed rest, or aging. The detached otoliths gather in the semicircular canals, and since each semicircular canal is spatially located on a different plane when the detached ear holes gather in one or more of them, they can become irritated and cause vertigo and nystagmus as they change position. It is common in elderly women in their 60s, and the recurrence is frequent, with a recurrence rate of 15% to 20%.^[10]^ BPPV cannot be diagnosed by advanced imaging and can be accurately diagnosed and treated solely on physical examination results. If left undetected and untreated, BPPV can also lead to reduced quality of life and falls, and is the leading cause of injuries and trauma-related hospitalizations in older adults.^[11]^

**Figure 3.**
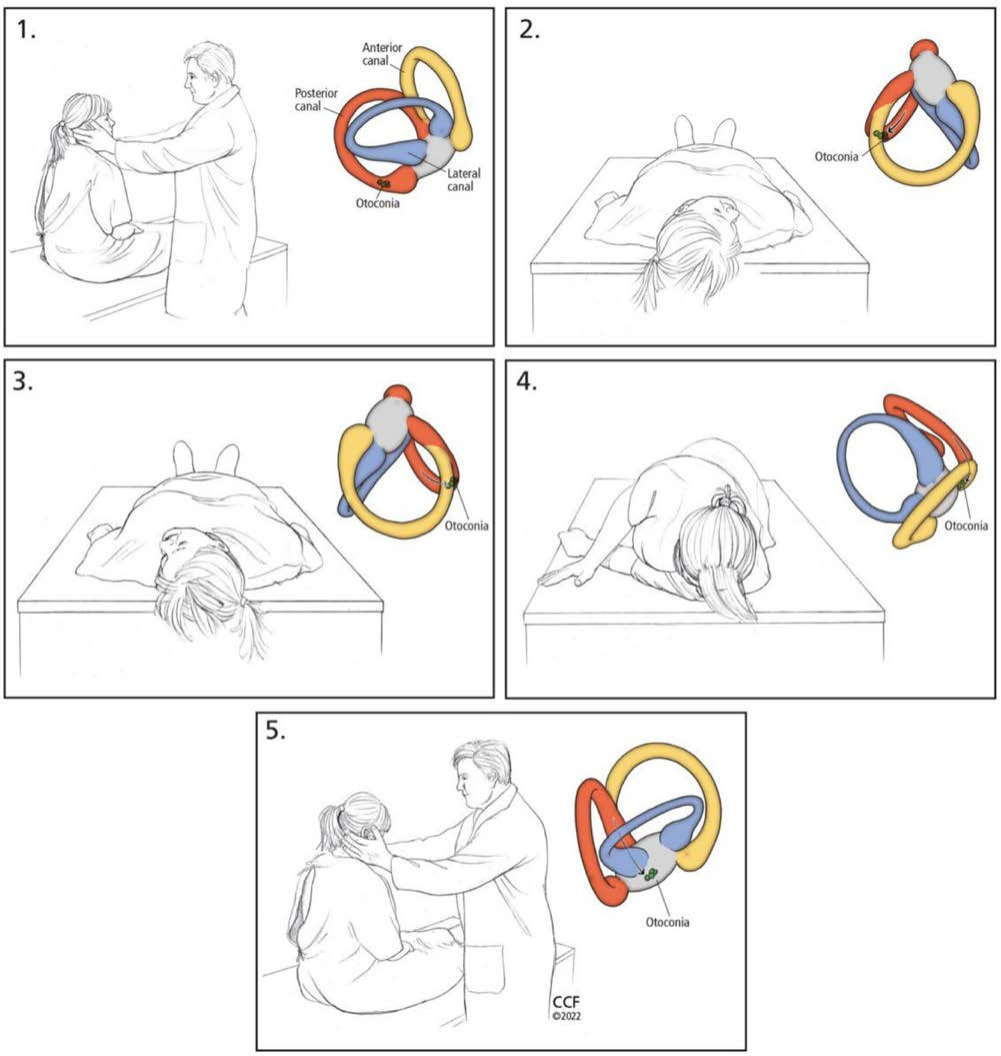
Epley manipulation: First, let the patient take the sitting position, rotate the head 45° to the affected side, and quickly move to the supine position; Then rotate the patient’s head to the other side by 45° and roll the patient to the healthy side, keeping the patient’s chin and ears at 45°. Finally, assist the patient to return to the sitting position and keep the direction of head rotation unchanged.

Using the Dix-Hallpike^[12]^ method as an examination-induced diagnostic technique, which is a test for BPPV affecting the vertical semicircular canal, the patient’s head is rotated 45° and then moved from a sitting position to supine position and from a supine position to a sitting position. The doctor will observe dizziness and horizontal nystagmus in the patient with each change of position. In 1992, Epley^[3]^ described a series of head and body movements to move otoliths out of the vertical semicircular canal through a series of body movements. Epley manipulative reduction mainly removes otolith from the posterior semicircular canal or anterior semicircular canal (Fig. 3).^[13]^ There are many reset techniques, but the principle is the same, to master the reset principle, flexible use. Manual reduction has been a relatively successful approach in the treatment of BPPV, but many patients have recurrent episodes, with a reported recurrence rate of about 50% during 10 years of follow-up,^[14]^ and frequent relapses cause much inconvenience to patients’ daily lives.

Among the non-vestibular vertigo diseases, CV has not been recognized by most doctors due to the lack of specific clinical symptoms and reliable diagnostic criteria. However, over the past 150 years, a large body of experimental and clinical evidence has accumulated to support CV as a separate disease name.^[15]^ CV is caused by cervical trauma, inflammation, degeneration, or mechanical dysfunction and is characterized by neck pain, stiffness, headache, dysphagia, nausea, visual impairment, ear distention, tinnitus, and temporomandibular joint pain.^[16]^ Many researchers have classified the causes of CV into 3 broad categories: sympathetic dysfunction, proprioceptive CV, vertebral artery rotation vertigo, and migraine-related vertigo.^[17]^ Research on CV has been plagued by the lack of a definitive diagnosis, lack of specific laboratory tests, and differences in the degree of vertigo among patients with neck pain.^[18]^ At present, there is no dispute about vertebral artery rotatory vertigo. Lesions in the upper neck segment lead to abnormal vertebral artery blood supply and insufficient vertebrobasilar artery blood supply, leading to dizziness, headache, nausea, tinnitus, and other symptoms. The most likely causes of abnormal vertebral artery blood supply in the upper cervical segment are atlantooccipital joint and atlantoaxial joint. The suboccipital muscle attached between the 2 joints controls the flexion, extension, and rotation of the joint. The suboccipital muscles include the posterior rectus capitis major, the posterior rectus capitis minor, the obliquus capitis, and the inferior obliquus capitis, of which the inferior obliquus capitis is connected to the atlantoaxial joint. Some studies believe that the main role of this short muscle is to stabilize the spine and control posture, and the abnormality of the suboccipital muscle is closely related to headache and neck pain.^[19]^ The subcutaneous nerves distributed in the posterior occipital area, including the greater occipital nerve, the lesser occipital nerve, the third occipital nerve, and the greater auricular nerve, all pass through the Atlantean, the axial vertebrae, the occipital bone, and the surrounding muscles. Due to joint dislocation and muscle spasm, the subcutaneous nerves are trapped during the perforation, resulting in symptoms such as paresthesia, pain, and numbness in the subcutaneous nerve distribution area.

Some studies have suggested that Atlantic Rotary Fixation (AARF) is the main cause of CV.^[20]^ AARF refers to any deviation from the normal rotation of the atlantoaxial complex in the upper cervical spine due to traumatic or non-traumatic causes.^[21]^ The treatment of AARF mainly includes conservative manipulation and invasive surgery. Due to the special anatomy of the atlantoaxial joint, AARF causes varying degrees of torsion and spasm in the muscles, nerves, and blood vessels attached to c1 and c2. Among them, the vertebral artery is seriously affected. In the occipital segment, the vertebral artery is out of shape with 5 twists and turns. When the local tissue spasms and degeneration, the vertebral artery is pulled and entrapment, resulting in poor blood flow, which leads to insufficient blood supply to the brain and dizziness. Some researchers^[22]^ have studied the relationship between rotational fixation of the atlantoaxial joint and vertebral artery and concluded that when the atlantoaxial joint is rotated clockwise and fixed relative to the axial vertebra, the second curvature of the occipital segment of the right vertebral artery and the vertebral segment between the 3 cervical bodies are stretched and trapped, and the second curvature of the occipital segment of the left vertebral artery and the entire vertebral segment is stretched and trapped. When the cervical spine is in the cervical rotation position, normal people will not cause insufficient blood supply due to sudden changes in the position of the head and neck. When people turn their heads, one side of the vertebral artery is distorted due to the regulation of neurohumoral fluid, and the other side of the vertebral artery can play a compensatory role so that the blood flow quickly returns to normal. However, if one side of the vertebral artery cannot supply blood normally due to the surrounding tissue compression, stimulation, and other reasons, then the other side of the vertebral artery is distorted by neck rotation, and both sides of the vertebral artery cannot supply blood normally, resulting in a decrease in blood flow and a series of symptoms after cerebral ischemia. This can also explain why CV patients in certain positions, such as turning their heads, symptoms will be aggravated.

The reduction principle of tuina manipulation is to adjust the position of the atlantoaxial joint, restore the mechanical balance of the cervical spine, reduce the stimulation of muscles and blood vessels, and thus relieve vertigo. Tuina combined with atlantoaxial directional inverted reduction technique use of the functional anatomical theory of atlantoaxial joint, with small range, light force, and no instantaneous cervical rotation, manual action can improve the dislocation of atlantoaxial joint, adjust the micro-displacement of atlantooccipital joint, relieve the pulling and entrapment of vertebral artery in atlantoaxial and occipital segments, and restore the dynamic balance of the cervical spine.

As the reviewer suggested the study has potential limitations. For CV with BPPV caused by rotational fixation of the atlantoaxial joint, manipulation, and reduction are effective. There are many causes of vertigo, and the content of this study is relatively limited. Future research will expand the scope of research to address more vertigo diseases.

## 4. Conclusion

To sum up, this case was composite vertigo caused by manipulation for 2 reasons, including CV caused by rotational fixation of the atlantoaxial joint treated by tuina manipulation and BPPV treated by reduction manipulation. It proves the effectiveness of massage manipulation and reduction manipulation and also proves the importance of diagnosis and differential diagnosis. There are many causes of vertigo, according to the inquiry, physical examination combined with auxiliary examination, to exclude emergency diseases, and treatment one by one.

## Acknowledgments

The authors would like to thank the patient and his guardians.

## Author contributions

**Conceptualization:** Yahui Sun.

**Resources:** Xingquan Wu.

**Writing – original draft:** Yahui Sun, Huijuan Lou.

**Data curation:** Jinglei Jiang, Zhongxu Li.

**Supervision:** Yiping Sun, Jing Xu.

**Writing – review & editing:** Deyu Cong.
